# The effects of adding local infiltration analgesia of the knee to a multimodal pain protocol for total arthroplasty: A matched pair retrospective study

**DOI:** 10.1080/24740527.2019.1603077

**Published:** 2019-04-29

**Authors:** Asher Selznick, Tejinder Chhina, Vir B. Sennik, Kenny Tam, Hossam El Beheiry

**Affiliations:** a Department of Anesthesia, Trillium Health Partners, Mississauga, Ontario, Canada; b Department of Anesthesia, University of Toronto, Toronto, Ontario, Canada; c Department of Orthopedic Surgery, Trillium Health Partners, Mississauga, Ontario, Canada; d Department of Surgery, University of Toronto, Toronto, Ontario, Canada

**Keywords:** knee arthroplasty, postoperative pain, local infiltration analgesia, multimodal, opioids

## Abstract

**Background**: We hypothesize that the addition of local infiltration analgesia (LIA) to a multimodal pain protocol will reduce the total amount of opioids consumed for acute pain control post total knee arthrolplasty (TKA).

**Methods**: This study was a retrospective, matched pair study including patients who had primary TKA. All patients included in the analysis had preoperative oral celecoxib and acetaminophen, had single-dose spinal anesthetic with intrathecal morphine, and had intravenous patient-controlled analgesia with an opioid agent in addition to gabapentin and celecoxib in the first 48 h. Patients whose charts were excluded from the study had revision TKA, received opioid therapy prior to the surgery, were classified as American Society of Anesthesiology (ASA) IV, and had general anesthesia. Fifty patients who underwent TKA and had LIA were matched for age, body mass index (BMI), and gender with patients who did not receive LIA. The primary outcome measures were total doses of opioids consumed post TKA.

**Results**: Patients receiving LIA consumed on average significantly less intravenous (IV) morphine equivalents than patients not receiving LIA, with a mean difference (±SD) of 88.9 ± 15.6 mg IV morphine equivalents. Furthermore, pain control was better in the LIA group. The incidences of nausea and vomiting, pruritis, and excessive sedation were higher in the non-LIA group compared to the LIA group. There was no difference in the hospital length of stay between both groups.

**Conclusions**: The addition of LIA to our multimodal pain protocol for TKA was associated with a reduction in total opioid consumption.

## RÉSUMÉ

**Contexte**: Nous émettons l’hypothèse que l’ajout d’une analgésie locale par infiltration à un protocole de prise en charge de la douleur multimodal réduira la quantité totale d’opioïdes consommée pour maitriser la douleur aigue après une arthroplastie totale du genou (ATG).

**Méthodes**: Cette étude était une étude rétrospective appariée ont les participants étaient des patients ayant subi une ATG primaire. Tous les patients inclus dans l’analyse s’étaient vus administrer du célécoxib et de l’acétaminophène en période préopératoire, une dose unique d’anasthésiant rachidien sous forme de morphine intrathécale et un analgésique intraveineux contrôlé par le patient comprenant un agent opioïde, en plus de la gabapentine et de l’acélécoxib au cours des premières 48 heures. Les patients dont les dossiers ont été exclus de l’étude avaient subi une ATG de reprise, avaient consommé des opioïdes avant la chirurgie, étaient classés ASA IV et avaient subi une anesthésie générale. Cinquante patients ayant subi une ATG et une analgésie locale par infiltration ont été appariés selon l’âge, l’IMC et le genre avec des patients qui n’avaient pas reçu d’analgésie locale par infiltration. Les résultats primaires ont été mesurés par les doses totales d’opioïdes consommées après l’ATG.

**Résultats**: En moyenne, les patients ayant reçu une analgésie locale par infiltration ont consommé significativement moins d’équivalents morphine IV que les patients qui n’avaient pas reçu d’analgésie locale par infiltration, avec une différence moyenne (± É.-T.) de 88,9 ± 15,6 mg d’équivalents morphine IV. De plus, la douleur était mieux maitrisée dans le groupe ayant reçu une analgésie locale par infiltration. L’incidence des vomissments, du prurit et de la sédation excessive était plus élevée dans le groupe n’ayant pas reçu d’analgésie locale par infiltration que dans le groupe ayant reçu une analgésie locale par infiltration. Il n’y avait pas de différence entre les deux groupes en ce qui concerne la durée du séjour à l’hôpital.

**Conclusions**: L’ajout d’une analgésie locale par infiltration à notre protocole douleur multimodal pour l’arthroplastie totale du genou était associé à une réduction dans la consommation totale d’opioïdes.

## Introduction

Total knee arthroplasty (TKA) is associated with significant immediate postoperative pain in the majority of patients. Nearly half of patients undergoing TKA experience extreme acute pain.^1^ This can lead to increased opioid consumption and delayed rehabilitation as well as development of chronic pain in about 20% of patients.^1^ Furthermore, severe pain can lead to an increasing incidence of thromboembolism because of immobility and cardiac events due to increased body stress responses.^2^ Therefore, many investigations attempted to identify the most effective protocol to control acute postoperative pain in TKA. However, the best protocol to control in hospital pain post TKA has not been standardized and its features are continuously changing.^1,3,4^ For example, epidural analgesia and single-dose or continuous femoral nerve blockade usage have been steadily declining because of the increased incidence of motor weakness and consequently deferred rehabilitation. Additionally, intravenous patient-controlled analgesia (PCA) is frequently replaced with oral PCA to decrease the incidence of opioid complications, particularly respiratory depression and arrest. Furthermore, the concept of a multimodal protocol including multiple measures for pain control has become more popular to provide better effectiveness and decrease side effects of individual components.^5^ These components currently include intrathecal opioid, oral PCA with opioids, and nerve blockade, particularly adductor canal block, which does not cause quadriceps motor weakness.^6^

In recent years, there has been an increasing interest in adding local infiltration analgesia (LIA) of the knee as a major component of multimodal acute pain relief protocol.^7^ Despite the positive experience with LIA, it is still being evaluated for its clinical utility. Therefore, the objective of this retrospective study is to elucidate the role and value of adding LIA to post TKA pain control protocol. We hypothesize that the addition of LIA to a multimodal pain protocol including intrathecal preservative-free morphine and intravenous opioid PCA reduces the total amount of opioids consumed for immediate pain management post TKA. Total opioid consumption in morphine equivalents has been considered a surrogate end point representing the efficacy of LIA for pain control; that is, the less morphine equivalents consumed the more efficacious the addition of LIA.

## Materials and methods

This study was a retrospective, single-center, multisurgeon, matched-pair study including patients who had primary TKA in the period including 2015 and 2016. The study was conducted at a tertiary health care facility after appropriate research ethics board approval.

The charts of 270 patients who had primary TKA were reviewed retrospectively. Charts were reviewed in sequence from the beginning of 2015 to the end of 2016. There was no specific temporal sequence for LIA and non-LIA patients because knee replacements were done by all surgeons simultaneously. Patients whose charts were included in the retrospective study had primary TKA, were ≥21 years of age at time of surgery, were classified as ASA I to III, and received or did not receive LIA. In addition, all patients included in the study received spinal anesthesia with hyperbaric bupivacaine 0.75% (1.5–2 ml) with intrathecal morphine (150 μg) as well as the other components of a standardized multimodal pain protocol, including preoperative oral celecoxib and acetaminophen, intravenous PCA with an opioid agent (hydromorphone), and oral gabapentin and celecoxib in the first 48 h. Patients whose charts were excluded from the study had revision TKA, received opioid therapy in any form prior to the surgery, were classified as ASA IV, had general anesthesia, or did not receive the multimodal pain protocol mentioned above.

The charts of eligible patients were reviewed in detail. Data were extracted from electronic medical records of the Trillium Health Partners information system and a database kept in the orthopedic department. The chart review was performed by a single person who was not involved in the analysis of the results. The investigator analyzing the results was blinded to the groups. Additionally, all surgeons who performed the knee replacements were not involved in the data collection or the statistical analysis of the results. Patients ≥21 years of age who underwent primary TKA and had LIA (LIA group) were matched for age, body mass index (BMI), and gender with patients who underwent primary TKA who did not receive LIA (non-LIA group). The matching ratio was 1:1 for LIA; that is, the total sample size was 100 patients. Age and BMI were matched based on 5-year (±2.5 years) and 5-kg/m^2^ (±2.5 kg/m^2^) intervals respectively. Age, BMI, and gender were chosen for matching because they were shown to affect the prescription of opioids after surgery.^8^

Patients in this cohort had similar surgical interventions. The thigh tourniquet inflation pressure was twice the patients’ preoperative systolic blood pressure and the duration of the tourniquet did not exceed 110 min. They had a medial parapatellar incision without any drains inserted. Tranexamic acid 20 mg/kg was administered intravenously after tourniquet deflation. Wound closure for all cases was performed with absorbable and nonabsorbable sutures at the level of the arthrotomy and absorbable sutures in the subcutaneous layer. The skin was re-approximated with either staples in most patients (about 90% in both groups) or a running subcuticular absorbable suture with steristrips. The dressing of the wound consisted of an inner layer of soft padding surrounded by a layer of short stretch compressive bandage applied firmly from the mid-calf to mid-thigh. All postoperative interventions undertaken were standard of care for our facility and were identical for each patient, including wound dressings and their removal, deep vein thrombosis prophylaxis, and postoperative rehabilitation. Postoperative pain assessed in this study was performed by two registered nurses who were trained to be members of the acute pain service in our institution. They used a standard preprinted 10-cm vertical visual analog scale (VAS) for all patients. Pain was measured at rest with knee in the neutral position and during passive knee flexion to 90°.

The primary outcome measures were total opioid consumption including opioids consumed from the intravenous PCA pump and after discontinuation of the PCA pump. All opioids consumed were converted into intravenous (IV) morphine equivalents (Appendix 1). The secondary outcome measures were details of surgical intervention, resting VAS, dynamic VAS during bending the knee by 90°, rate of postoperative surgical infection, time to start rehabilitation after surgery, length of hospital stay, occurrence of nausea and/or vomiting, pruritis, sedation, hallucination, hypotension, respiratory depression, and respiratory arrest. Other confounders and co-interventions included patient demographics, preoperative medications, preoperative nonopioid analgesics, postoperative nonopioid analgesics, comorbidities, surgeon’s name, surgical duration, and type of prosthesis.

### Technique of LIA

**Figure 1.**
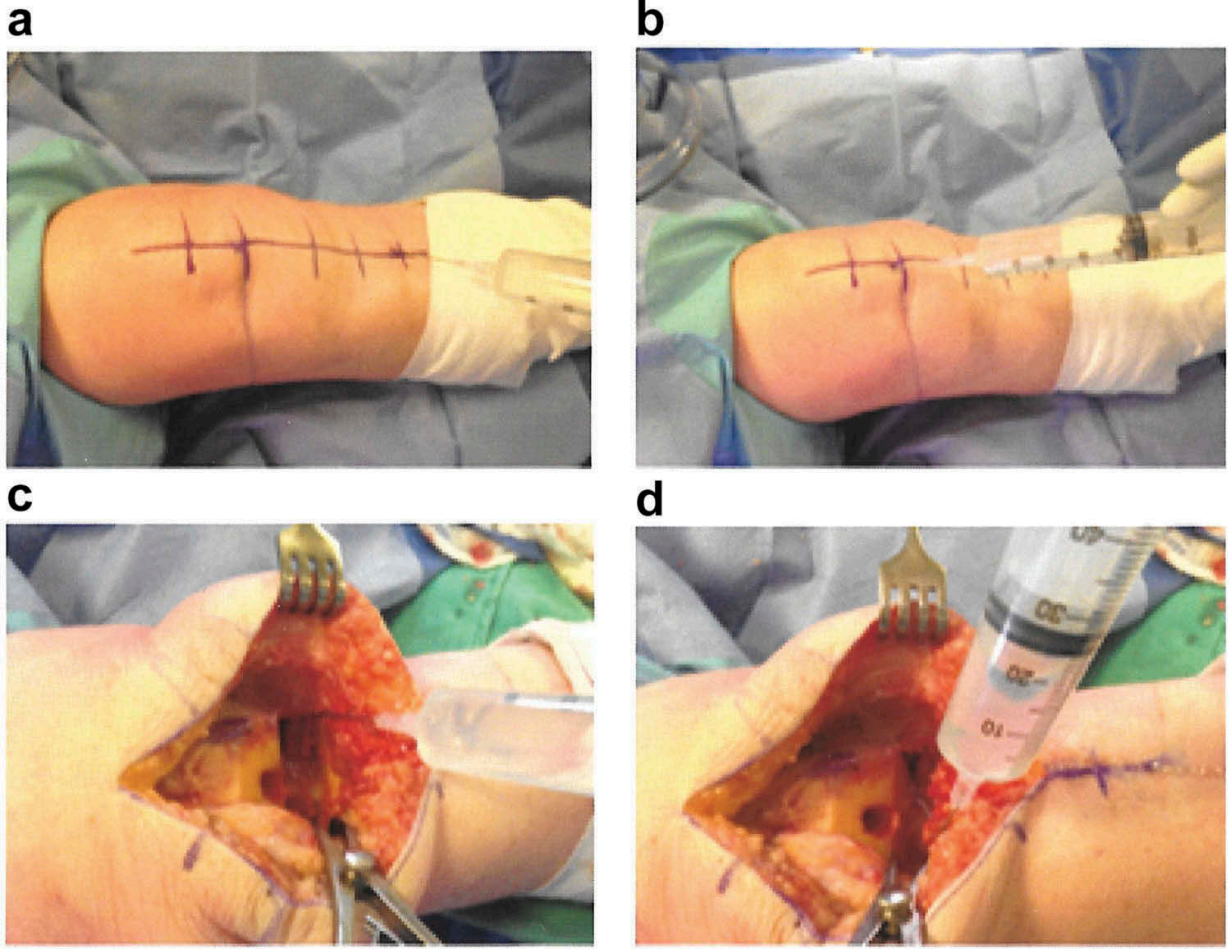
The technique of local infiltration analgesia during total knee arthroplasty. (a) Infiltration of the skin incision. (b) Infiltration proximal to the knee joint to block the intermediate and medial cutaneous nerves of the thigh and in areas of fat deep to the fascia. (c), (d) Infiltration of the medial and lateral aspects of the posterior capsule.

The injection mixture was the same for all patients included in this study. The injection mixture consisted of bupivacaine 0.25% (40 ml), preservative-free morphine 5 mg (10 ml), ketorolac tromethamine 30 mg (1 ml), and epinephrine 0.3 mg (0.33 ml). The mixture was made up to 60 ml with normal saline. Strict sterility precautions were implemented. Local infiltration analgesia was achieved by periarticular infiltration of the knee joint. First, before the surgical incision, one third of the mixture was injected along the anticipated skin incision, proximal to the knee joint, to block the intermediate and medial cutaneous nerves of the thigh and in areas of fat deep to the fascia. Second, following bone resection and prior to cementing the prosthesis, the posterior aspect of the joint was exposed and two thirds of the mixture was injected into the posterior capsule on each side (Figure 1).

### Statistical analysis and sample size calculation

Statistical analysis included the comparison between the non-LIA and LIA groups. Age, BMI, surgical duration, tourniquet time, length of hospital stay, and opioids consumed were compared using paired two-tailed Student’s *t*-test. Gender ratio, ASA classification, Zimmerman versus Johnson & Johnson prosthesis, incidence of pre-emptive analgesia, incidence of postoperative complications, and VAS scores were compared using Wilcoxon matched pairs signed rank test.

The sample size estimate for the patient charts to be reviewed was based on the difference in the primary outcome (i.e., total postoperative opioid consumption) among patients who had LIA and those who did not have LIA. Reviewing the available data and our clinical experience showed that LIA may produce a savings of up to 25% of the postoperative mean opioid consumption.^7,9,10^ Based on this information, the required sample size of the study was estimated to be 47 pairs of patient (paired *t*-tests: effect size f = 0.36, alpha = 0.05, one-tailed power = 0.8). The sample size was increased by about 10%, resulting in 50 pairs; that is, a total of 100 patients. These 100 patients were chosen from the 270 patients based on the inclusion and exclusion criteria for the study.

## Results

All of the primary TKAs were performed by four orthopedic surgeons. One surgeon was responsible for administering LIA to the LIA group. The other three surgeons were involved in performing the TKA in the non-LIA group. Each of the four surgeons had at least 15 years experiences as a senior consultant. Both groups had similar demographics indicating adequate matching (Table 1). All study patients in both groups had the same multimodal pain protocol except that patients in the LIA group had the addition of LIA performed intraoperatively (Table 2). The surgical duration was slightly less in the LIA group (Table 1). Furthermore, the addition of LIA was not associated with a change in the length of hospital stay (Table 1).

**Table 1.** Characteristics of cross-matched patients who had total knee arthroplasty.^a^

	Non-LIA (control) (*n* = 50)	LIA (*n* = 50)	*P* value^b^
Age (years)	70 ± 6	70 ± 6	0.763
Female/male	38/12	38/12	
ASA (I/II/III)	2/15/33	2/18/30	0.156
Body mass index (kg/m^2^)	31 ± 5	30 ± 5	0.06
Duration of surgery (min)	72 ± 16	61 ± 7	0.0001
Tourniquet time (min)	65 ± 17	61 ± 7	0.167
Pre-emptive analgesia^c^ (yes/no)	42/8	40/10	0.795
Type of prosthesis (J&J/Z)	24/26	25/25	0.500
Length of hospital stay (days)	4 ± 2	4 ± 1	0.165

^a^Data are presented in proportion or mean ± SD.

^b^P value ≤ 0.05 is considered statistically significant.

^c^Pre-emptive analgesia indicates the administration of oral Celebrex and Tylenol about 60 min prior to surgery.

LIA = local infiltration analgesia of the knee joint; J&J = Johnson & Johnson prosthesis; Z = Zimmerman prosthesis.

**Table 2.** Multimodal pain protocol implemented in the study patients (*n* = 100).

	Dose	Route	Frequency
Preoperative			
Acetaminophen	1000 mg	Oral	Single dose
Celecoxib	400 mg	Oral	Single dose
Gabapentin	200 mg	Oral	Single dose
Intraoperative			
Morphine (preservative free)	150 µg	Intrathecal	Single dose
LIA^a^		Intra-articular	Single dose
Postoperative			
PCA^b^	0.2 mg	Intravenous	Lock interval 8–10 min
Celecoxib	400 mg	Oral	Every 12 h for 2 days
Gabapentin	200–300 mg	Oral	Every 8 h for 2 days
Acetaminophen	650 mg	Oral	Every 6 h for 2 days
Opioids (ad libitum)			
Hydromorphone	0.2–0.4 mg/1–2 mg	IV/PO	Every 4 h as needed
Morphine	2–4 mg/10–20 mg	IV/PO	Every 4 h as needed
Tramadol	50 mg	Oral	Every 6 h as needed
Oxycodone	10–20 mg	Oral	Every 4 h as needed

^a^LIA was given to 50 patients. Each of these patients was matched to a non-LIA patient. The LIA mixture contained bupivacaine 0.25% (40 ml), preservative-free morphine 5 mg (10 ml), ketorolac tromethamine 30 mg (1 ml), and epinephrine 0.3 mg (0.33 ml). The mixture was made up to 60 ml with normal saline.

^b^PCA hydromorphone was used in all patients included in the study.

LIA = local infiltration analgesia; PCA = patient-controlled analgesia; IV = intravenous; PO = by mouth.

Daily opioid consumption through the PCA pump and non-PCA pump was higher in the non-LIA group compared to the LIA group (Table 3). Total opioid consumption was defined as the consumption of IV and oral opioids including IV PCA opioids on the day of surgery and during the initial three postoperative days. The mean difference in the total opioid consumption between the non-LIA and LIA groups was 88.9 ± 15.6 mg IV morphine equivalents, indicating significantly less total opioid consumption in the LIA group (Table 4).

**Table 3.** Postoperative opioid consumption in IV morphine equivalent (mg) in the LIA and non-LIA groups.

	Non-LIA		LIA	
	PCA opioids	Non-PCA opioids	PCA opioids	Non-PCA opioids
POD 0	19.64 ± 3.93*	0.00 ± 0.00	3.85 ± 1.31	0.00 ± 0.00
POD 1	84.84 ± 21.28*	35.98 ± 8.2*	47.71 ± 19.08	27.81 ± 5.65
POD 2	10.94 ± 3.4*	49.42 ± 17.3*	2.29 ± 0.58	36.98 ± 7.4
POD 3	0.00 ± 0.00	28.98 ± 5.79	0.00 ± 0.00	22.19 ± 5.33
Total	115.4 ± 49*	114.4 ± 36.9*	53.9 ± 25	87.0 ± 33

*P ≤ 0.05 between non-LIA and LIA groups.

IV = intravenous; LIA = local infiltration analgesia; PCA = patient-controlled analgesia; POD = postoperative day.

**Table 4.** Total opioid consumption during the initial three postoperative days in morphine IV equivalents (mg).

	Total PCA consumption	Total non-PCA consumption	Total opioid consumed
Non-LIA (*n* = 50)	115.4 ± 49^a^	114.4 ± 36.9^a^	229.8 ± 80.4^a^
LIA (*n* = 50)	53.9 ± 25	87.0 ± 33.0	140.9 ± 66.4

^a^Indicates statistically significant (*P* ≤ 0.05) difference from the LIA group.

IV = intravenous; PCA = patient-controlled analgesia; LIA = local infiltration analgesia of the knee joint.

There was no relationship between total opioid consumption and the date of surgery for patients in both groups as depicted by Spearman’s rank order correlation coefficient (*r_s_*). This indicates that the lack of matching of cases and controls for the date of procedure has no effect on the outcomes of the study. The *r_s_* for the correlation between total opioid consumption and the surgical date for the non-LIA and LIA groups was 0.09 (*P* = 0.294, two-tailed) and −0.05 (*P* = 0.29, two-tailed), respectively. Additionally, there was no statistical difference in total opioid consumption between patients of the three surgeons who did not perform LIA.

**Figure 2.**
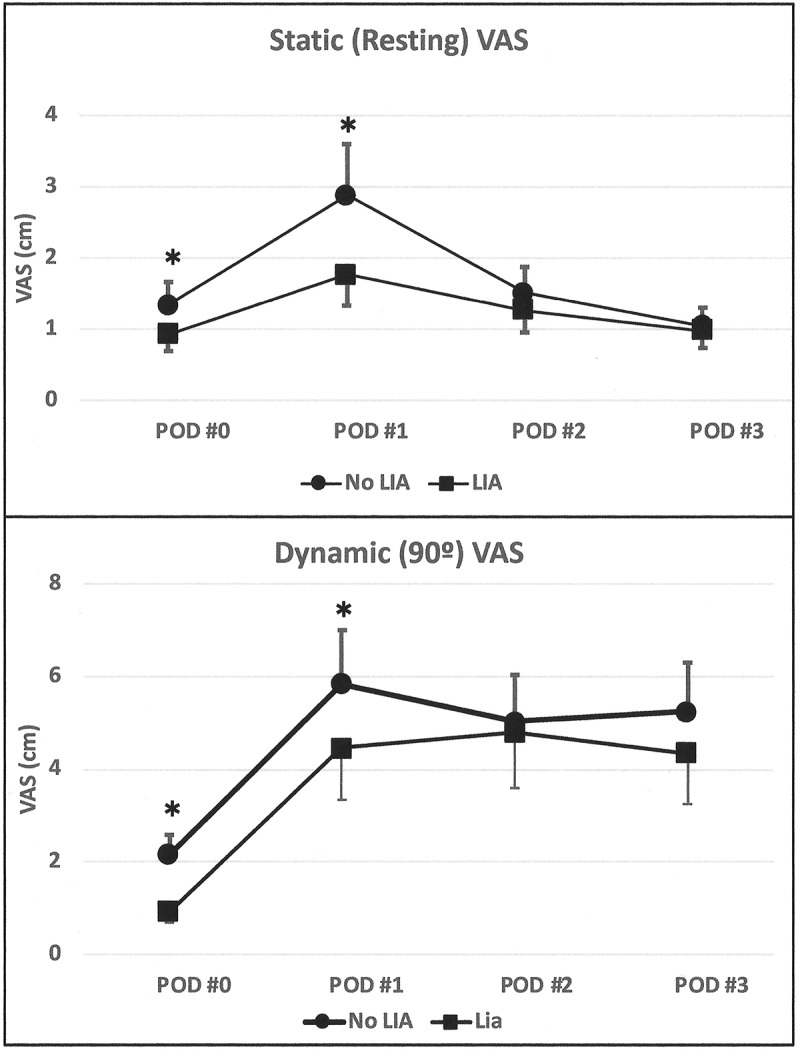
Static (resting) and dynamic (90° knee flexion) postoperative VAS scores. The figure shows the initial VAS evaluated by the acute pain service about 16 h after discharge from the postanesthesia recovery unit and subsequent daily assessment. POD indicates postoperative day. *P ≤ 0.05.

The VAS for pain was significantly lower in the LIA group compared to the non-LIA group during the day of surgery and the first postoperative day (Figure 2). The resting and dynamic knee VAS scores were consistently higher on the day of surgery and the first postoperative day in the non-LIA group (Figure 2). In contrast, the resting and dynamic VAS were similar in both groups on the second and third postoperative days.

The incidences of nausea and vomiting, pruritis, and excessive sedation were higher in the non-LIA group compared to the LIA group (Table 5). This is probably due to the side effects of increased total opioid consumption in the non-LIA patients. Interestingly, other side effects of opioids including the frequency of respiratory depression, respiratory arrest, or hallucination were not different between the two groups (Table 5).

**Table 5.** Incidence of postoperative complications during hospital admission.^a^

	Non-LIA	LIA	*P* value
Surgical infection	0/50 (0.00%)	0/50 (0.00%)	N/A
Nausea and vomiting	38/50 (76%)	29/50 (58%)	0.046
Pruritis	8/50 (16%)	1/50 (2%)	0.014
Excessive sedation^b^	4/50 (8%)	0/50 (0.0%)	0.02
Hallucination	0/50 (0.00%)	0/50 (0.00%)	N/A
Hypotension	5/50 (10%)	4/50 (8%)	0.725
Respiratory depression	0/50 (0.00%)	1/50 (2%)	0.315
Respiratory arrest	0/50 (0.00%)	0/50 (0.00%)	N/A
Naloxone administration	0/50 (0.00%)	1/50 (2%)	0.315

^a^*P* value ≤ 0.05 is considered statistically significant.

^b^Excessive sedation was measured using the Fisher sedation score (0 = no sedation [alert patient], 1 = mild sedation [patient drowsy, open eyes to verbal stimulation], 2 = moderate sedation [patient drowsy, open eyes to tactile stimulation], 3 = severe or excessive sedation [patient somnolent, difficult to arouse by verbal or tactile stimulation], S = normal sleep). Sedation was recorded every 2 to 4 h after discharge from the postanesthesia recovery unit until the discontinuation of the patient-controlled anesthesia pump.

LIA = local infiltration analgesia of the knee joint.

## Discussion

The results of this study support our hypothesis that the addition of LIA to a multimodal pain protocol reduces opioid consumption in the days following TKA. Patients receiving LIA consumed on average significantly less opioids compared to patients not receiving LIA, with a mean difference (±SD) of 88.9 ± 15.6 mg IV morphine equivalents. Furthermore, pain control as measured by VAS was better in the LIA group. The resting and dynamic knee bending 90° VAS scores were consistently higher on the day of surgery and the first postoperative day in the non-LIA group.

The precise components of the LIA mixture have yet to be standardized between institutions and even between orthopedic surgeons. In fact, randomized controlled trials to address this issue should be performed to reach definitive conclusions about the efficacy of each component of the LIA mixture. However, LIA mixture has been always reported to contain a combination of local anesthetic, opioid, anti-inflammatory drug, and vasoconstrictor. The total volume and technique of injection is also varied across studies, hospitals, and surgeons. This makes direct comparison of different studies evaluating LIA somewhat difficult, yet patterns and conclusions can still be drawn. For example, a systematic review including 21 articles published between 2006 and 2011 as well as a recent study showed that LIA achieved superior pain relief compared to exclusive intravenous analgesia.^9,10^ More recently, LIA was found to provide more efficacious pain relief compared to placebo.^7^ In addition, LIA compared to standard analgesia, including femoral and sciatic nerve blocks, resulted in greater pain relief and improvement in range of motion.^11^ Despite the above trend of positive results suggesting the superiority of the LIA technique, other previous reports concluded that blocking multiple nerves was preferable or at least comparable to LIA, particularly adductor canal block.^12–15^ Our study supports the evidence that LIA added to a multimodal pain protocol without peripheral nerve blocks (Table 2) significantly lowers acute pain following total knee replacement surgery.

The LIA mixture used in our study consisted of a combination of bupivacaine (100 mg), preservative-free morphine (5 mg), ketorolac tromethamine (30 mg), and epinephrine (0.3 mg),^16^ made up to 60 ml with normal saline. The agents used have a synergistic action that attenuates many mechanisms of acute postoperative pain. The local anesthetic blocks sodium channels and sensory impulses along the peripheral nerves and nerve endings exposed by the surgical procedure. Epinephrine vasoconstricts blood vessels and thus prolongs the action of the local anesthetic by decreasing absorption.^16^ Additionally, epinephrine reduces intra-articular bleeding and postoperative hematoma.^16^ Morphine has a local pain modulating effect by stimulating peripheral opioid receptors. Ketorolac reduces the inflammatory responses that enhance pain perception. Other added agents were reported in the literature but not used in this study cohort; for example, alpha-2 agonists (clonidine).^17^

Our study has a few limitations. First, the cases and controls were not matched for the date of procedure. However, there was no relationship between the extent of the primary outcomes of the study and the date of surgery in the whole cohort as depicted by the insignificant *r_s_* correlation coefficient between total opioid consumption and date of procedure in both groups. Second, the LIA group was limited to one surgeon, whereas three surgeons performed TKA in the non-LIA group. This may have led to sampling bias pertaining to the surgical technique and consequently the extent of postoperative pain. Nonetheless, all surgeons who performed the surgical procedures were senior consultants with more than 15 years of experience and used standardized techniques as well as the same type of knee prothesis. Third, there may have been possible variations in individual patient’s perioperative care, thus creating unavoidable sampling bias. However, all intraoperative and postoperative interventions followed standard protocols for our institution and were performed by a single team; that is, there was no specific team for each surgeon. This will largely decrease the variations in the individual patient’s perioperative care and consequently possible sampling bias. In keeping with this concept, the duration of surgery in the LIA group was about 15% shorter than that in the non-LIA group. Such a difference, though statistically significant, is of no clinical significance and the difference did not impact or interfere with patient care. Fourth, possible sampling bias was kept to a minimum pertaining to patient selection for the study because patient selection was based on inclusion and exclusion criteria as well as precise matching. Taken together, sampling bias in the present study is minimal because patient selection and matching followed clear criteria, surgical techniques and type of prostheses used were similar in both groups, all perioperative interventions for all patients included in the study followed institutional defined protocols, and patients were cared for by single team. It is unlikely that significant sampling bias could have had major effects on the results of this study.

In conclusion, LIA is a promising technique for reducing in hospital acute pain following TKA. The LIA technique has a minor impact on the workflow and on the operating room budget while providing significant benefit to patients. It decreases total opioid consumption and consequently their side effects, namely, nausea and vomiting, sedation, ileus, and urinary retention. Thus, patients will start rehabilitation quicker and will be discharged sooner. Future studies should aim to provide objective information pertaining to the optimal combination of the LIA mixture as well as its value when added to different multimodal acute pain management protocols.

## Appendix 1

IV morphine equivalent dose conversion table for opioids.^a^

**Table:**

Opioid	Route	IV morphine equivalent factor	Opioid dose equivalent to 10 mg IV morphine
Codeine	IM	0.1	100 mg
	PO	0.05	200 mg
Fentanyl	IV	0.1	100 µg
Hydromorphone	IV/IM/SC	5	2 mg
	PO	2	5 mg
Meperidine	IV/IM/SC	0.1	100 mg
	PO	0.05	200 mg
Morphine	IV/IM/SC	1	10 mg
	PO	0.4	25 mg
Oxycodone	PO	0.67	15 mg
Tramadol	PO	0.05	200 mg

^a^Data from Reddy et al.,^18^ Nielsen et al.,^19^ and Pereira et al.^20^

IV = intravenous; IM = intramuscular; PO = by mouth; SC = subcutaneous.
